# DR-ALOHA-Q: A Q-Learning-Based Adaptive MAC Protocol for Underwater Acoustic Sensor Networks

**DOI:** 10.3390/s23094474

**Published:** 2023-05-04

**Authors:** Slavica Tomovic, Igor Radusinovic

**Affiliations:** Faculty of Electrical Engineering, University of Montenegro, 81000 Podgorica, Montenegro; igorr@ucg.ac.me

**Keywords:** medium access control, reinforcement learning, underwater acoustic networks

## Abstract

Underwater acoustic sensor networks (UASNs) are challenged by the dynamic nature of the underwater environment, large propagation delays, and global positioning system (GPS) signal unavailability, which make traditional medium access control (MAC) protocols less effective. These factors limit the channel utilization and performance of UASNs, making it difficult to achieve high data rates and handle congestion. To address these challenges, we propose a reinforcement learning (RL) MAC protocol that supports asynchronous network operation and leverages large propagation delays to improve the network throughput.he protocol is based on framed ALOHA and enables nodes to learn an optimal transmission strategy in a fully distributed manner without requiring detailed information about the external environment. The transmission strategy of sensor nodes is defined as a combination of time-slot and transmission-offset selection. By relying on the concept of learning through interaction with the environment, the proposed protocol enhances network resilience and adaptability. In both static and mobile network scenarios, it has been compared with the state-of-the-art framed ALOHA for the underwater environment (UW-ALOHA-Q), carrier-sensing ALOHA (CS-ALOHA), and delay-aware opportunistic transmission scheduling (DOTS) protocols. The simulation results show that the proposed solution leads to significant channel utilization gains, ranging from 13% to 106% in static network scenarios and from 23% to 126% in mobile network scenarios.oreover, using a more efficient learning strategy, it significantly reduces convergence time compared to UW-ALOHA-Q in larger networks, despite the increased action space.

## 1. Introduction

Underwater acoustic sensor networks (UASNs) have attracted considerable attention over the past decade due to their potential to support a wide range of applications that can generate benefits for humans. UASNs can find their applications in underwater pollution monitoring, coastline protection, mineral detection, oil reservoir management, emergency operation support, mine reconnaissance, assisted navigation, etc. [1]. However, while potential UASN applications are exciting, the design of UASN communication protocols faces many challenges. The critical issues are the slow propagation speed of acoustic signals (∼1500 m/s), narrow bandwidth, and high bit error rate [2]. These inherent characteristics of the underwater acoustic channel result in a low fundamental capacity according to Shannon’s capacity theory [3].

Medium access control (MAC) protocols are responsible for coordinating data transmissions of sensor nodes to avoid collisions at the receiver. Among the aforementioned challenges, the slow propagation speed of acoustic signals in water is the main hurdle for MAC efficiency. Due to large propagation delays, the reception time of data packets depends not only on the packet transmission time but also on the transmitter–receiver distance [4]. This means that simultaneous transmissions may not collide at the receiver, while collision of non-simultaneous transmissions is possible—the so-called space-time uncertainty phenomenon. MAC protocols used in terrestrial radio networks (e.g., TDMA, CSMA, ALOHA, etc.) are designed under the assumption that propagation delays are negligible. Thus, when directly applied in UASNs, these protocols experience drastic performance drops.

MAC protocols are generally classified as contention-free or contention-based. Contention-free protocols are based on the idea of dividing communication channels in time (TDMA), frequency (FDMA), or code domain (CDMA). TDMA is the basis of the most well-established research on UASN MAC [5]. The proposed solutions are able to achieve high throughput by relying on centralized resource (time-slot) scheduling and the knowledge of global network information, including link propagation delays. However, in many practical UASNs, global network knowledge is not available, as it is difficult and costly to track in the dynamic underwater environment. Moreover, TDMA-based approaches require clock synchronization across the network, which is difficult to achieve underwater due to GPS unavailability. Contention-based protocols aim to minimize the probability of collisions while operating in a distributed setting. They are categorized into handshaking-free and handshaking-based. The former are only suitable for lightly loaded networks [6]. The latter solve the hidden-terminal problem by performing a handshaking procedure before data transmission. However, due to large propagation delays, handshaking seriously degrades acoustic channel utilization. Therefore, there is no one-suits-all MAC solution for UASNs. The goal that is being pursued is the development of a distributed bandwidth- and energy-efficient MAC protocol that supports asynchronous network operation and fits well in dynamic environments.

In this paper, we propose a novel reinforcement learning (RL)-based MAC protocol for UASNs to address the dynamicity and complexity of underwater acoustic channels. The proposed protocol treats the sensor nodes as asynchronous learning agents that can learn optimal transmission strategies through trial-and-error interaction with the environment. In this way, the nodes can continually adapt to topological and environmental changes without gathering detailed network information such as link propagation delays. Our study is inspired by the research reported in [3,7], where the authors proposed UW-ALOHA-Q-RL-based framed ALOHA protocol for UASNs. UW-ALOHA-Q is the transformation of ALOHA-Q (designed for terrestrial radio networks) that enables the asynchronous operation of UASN and improved channel utilization. However, UW-ALOHA-Q uses the stop-and-wait transmission approach, where time slots are dimensioned to accommodate data packet transmission, maximum round-trip delay, and acknowledgment (ACK) packet reception. The number of slots in the frame is determined based on a heuristic formula, the efficiency of which varies with the network radius and topology. Furthermore, in UW-ALOHA-Q, nodes adapt their frame start time independently of the slot learning process (using a random backoff mechanism). Therefore, the network often fails to converge, as we will show in our study. To address the mentioned limitations, we propose a new RL-based MAC algorithm referred to as delayed-reward ALOHA-Q (DR-ALOHA-Q) for UASNs, which uses reduces time-slot size and enables the nodes to transmit in every time slot, regardless of whether the rewards for the previous time slots are received or not. In this way, the nodes can learn to utilize the available bandwidth more efficiently compared to UW-ALOHA-Q because no time slots are wasted due to long propagation delays. As an additional measure to boost the channel utilization, we increase the action space of the learning algorithm such that nodes select not only a time slot within the frame but also a time offset for packet transmission within the selected slot.

The key contributions of the paper are summarized as follows:

- We define DR-ALOHA-Q as a framed-ALOHA MAC scheme that selects optimal time slots and time offsets for packet transmissions for UASN nodes. As we will show later in the paper, joint optimization of time-slot selection and transmission offset enables DR-ALOHA-Q to achieve high channel utilization at a reasonable convergence speed. Therefore, clock synchronization is not required;
- We formulate the MAC problem as a decentralized multiagent reinforcement learning problem with a delayed reward. A computationally efficient RL model is run independently on each sensor node, without requiring any information exchange among the nodes. The total number of network nodes is the only global information assumed to be known locally. To maximize the network throughput, DR-ALOHA-Q deviates from the usual RL approach with an immediate reward mechanism, which is not suitable for environments with long propagation delays.
- Hysteretic Q-learning [8] is applied to enhance the convergence properties of the algorithm;
- We evaluate the performance of DR-ALOHA-Q through a series of simulations and compare it with UW-ALOHA-Q, [3], DOTS [9], and CS-ALOHA [10] for a wide range of static and mobile network scenarios. Our results show that DR-ALOHA-Q achieves significant improvements in terms of channel utilization. Furthermore, even when considering higher channel utilization targets, DR-ALOHA-Q achieves faster convergence times than UW-ALOHA-Q.

The rest of this paper is organized as follows. Section 2 summarizes related work. In Section 3, we present the considered UASN scenario and present the DR-ALOHA-Q protocol design. Simulation results are presented in Section 4. Section 5 concludes the paper.

## 2. Related Work

The research community has developed numerous MAC solutions to address the difficulties encountered in underwater acoustic communication. These solutions can be classified into two categories: contention-free and contention-based. TDMA-based protocols, such as UW-FLASHR [11], ST-MAC [12], STUMP [13], TDA-MAC [5], and DQUA-MAC [14], are examples of contention-free MAC protocols commonly used in underwater sensor networks. However, these protocols rely on centralized scheduling, which can lead to excessive control overhead or delayed responses to network dynamics. In contrast, contention-based MAC protocols offer greater flexibility and responsiveness to dynamic network conditions.

Contention-based protocols are further divided into handshaking-based and handshaking-free protocols. Many handshaking-based approaches for UASNs are based on MAC solutions used in terrestrial networks. For example, DACAP [15] and slotted FAMA [16] require nodes to wait for control packets such as request to send (RTS), clear to send (CTS), and acknowledgments (ACKs) to propagate through the slow acoustic medium before establishing a communication link for data transmission, resulting in low channel utilization. Some approaches leverage large propagation delays to enable parallel transmissions of sensor nodes. For instance, MACA-U [17] and APCAP [18] exploit a long waiting time during handshakes for data transmissions. DACAP [15] uses different handshake lengths for different recipients based on the estimated distance between the nodes. DOTS [9] utilizes locally obtained information about the propagation delays towards one-hop neighbors to enable concurrent transmissions with low probability of collision. It provides stable throughput and fair medium access, even in cases of node mobility. Despite the many improvements, the performance of UASN handshaking-based protocols is fundamentally constrained by prolonged channel reservation procedures or/and the inefficiency of carrier sensing caused by slow propagation of control signals in comparison to similar protocols used in terrestrial radio systems.

In lightly loaded networks, there has been significant interest in handshaking-free protocols, such as simple Aloha [19]. Various variants of Aloha-based protocols have been proposed for UASNs, such as CS-ALOHA [10], which employs only carrier sensing and an ACK mechanism; ALOHA-AN [20], which uses additional control packets for collision avoidance; and time-slotted variants that use increased guard times (S-ALOHA [21]) or transmit at a specific time that has been carefully calculated to synchronize the arrival time of the data packet with the beginning of the time slot at the receiver (SA-ALOHA [22]). All of these protocols suffer from low throughput in heavy traffic conditions. T-Lohi [23] follows a different approach whereby short contention signals (3B long tones) and predefined waiting times are used to resolve potential collisions. However, when many nodes compete for resources, the time it takes for one node to win the contention is likely to significantly exceed the duration of the actual data transmission.

In recent research studies, RL has been used for MAC in UASNs [3,7,24,25,26,27,28]. The advantage of learning-based MAC stems from its ability to accomplish and retain a (semi)optimal transmission strategy in a dynamic environment. Furthermore, learning-based algorithms can be typically implemented in a decentralized manner, and their communication overhead can be limited. All of these are desirable features for resource-limited networks operating in a highly unpredictable underwater environment. Solutions presented in [24,25,26,27] address specific scenarios that are not similar to that considered in this paper. In particular, the authors of ref. [24] used slotted carrier-sensing multiple access (slotted CSMA) and an RL algorithm to transmit packets over the optimal relay node and subchannel to the sink. The authors aimed to maximize the lifetime of the network as their primary optimization objective. However, the protocol requires time synchronization, which is difficult to achieve underwater, as well as periodic exchange of control packets for neighbor discovery, which may result in excessive energy consumption. The protocol design also incorporates multichannel communication, which is not suitable for UASNs in which the channel bandwidth is very limited. On the other hand, due to large propagation delays, CSMA is generally ineffective in UASNs, as it demands excessive guard intervals. In [26], the problem of transmission power control for collision avoidance was considered. The time-slotted contention-based MAC protocol proposed in [25] uses Q-learning to select the backoff waiting time before the packet transmission. The slot duration corresponds to the packet transmission time plus the guard interval. Improvements over UW-ALOHA-Q and MACA-U in terms of energy efficiency, channel utilization, and latency are proven via simulation analysis but under some very demanding assumptions. Namely, the protocol assumes that the nodes are synchronized, propagation delays between communicating nodes are known, nodes know the exact transmission times that correspond to data packet arrival at the beginning of the slot at the sink, etc. The solution proposed in [27] is handshaking-based and concerns the problem of multimedia streaming in multihop networks.

A learning-based MAC protocol designed for scenarios similar to those considered in this paper is UW-ALOHA-Q [3]. Compared to ALOHA-Q [29], which is designed for terrestrial radio networks, UW-ALOHA-Q incorporates three enhancements that make it more suitable for underwater environments: asynchronous operation, increased channel utilization through a reduced number of slots per frame, and a new random backoff scheme. The ability of the protocol to support mobility is demonstrated for a wide range of scenarios in [7]. However, the slots are designed to accommodate data and ACK packet transmission, maximum round trip time, and guard interval, whereas the optimal number of slots per frame is determined based on a heuristic formula taking the network radius and the number of nodes as input. As a result, the protocol exhibits very different behavior in different network configurations. Moreover, nodes use an uncoordinated random backoff mechanism to adjust the frame start time after several consecutive collisions. In larger networks, this significantly prolongs the convergence process to the optimal transmission strategy, as observed in Section IV. The problem of ALOHA-Q’s large overhead with respect to the slot size is tackled in [28], but the proposed solution is applicable to linear chain networks only.

Recently, deep reinforcement learning (DRL) has been applied in the MAC design for UASN [30,31]. In [30], a DRL mechanism with delayed reward and a nimble training mechanism was used nodes to determine the optimal transmission strategy in a mixed environment in which nodes with different protocols (such as TDMA and ALOHA) coexist. The authors also showed that the proposed solution outperforms slotted FAMA and DOTS in a homogeneous environment. A similar approach was proposed in [31] for hybrid optical and acoustic underwater sensor networks. The difficulty in applying DRL to MAC design is that the state space can be very large. In a network of *N* nodes, the state space is at least $N^{m}$ when accounting for the past *m* transmissions. In general, the limitations of the DRL approach include increased complexity, a long convergence time, and increased computation demand for each node. In [30], a network with only four DRL nodes was considered, and even then, the adaptation time was on the order of several thousand time slots.

The protocol proposed in this paper (DR-ALOHA-Q) is a handshaking-free contention-based protocol. Similar to [3,7], it is based on framed ALOHA and uses RL to enable nodes to discover optimal transmission strategies in a dynamic underwater environment, without requiring knowledge of global network information (e.g., propagation delays). Moreover, the protocol supports asynchronous operation, does not employ carrier sensing, and is characterized by very low complexity. In contrast to [3,7] but similar to [30], we consider a scenario in which the time-slot duration is far shorter than the propagation delay. Aiming to maximize the channel utilization under space-time uncertainty, the nodes are trained to select optimal time slots in the frame and to optimally delay packet transmission with a time slot.

## 3. Scenario and Network Model

**Figure 1:**
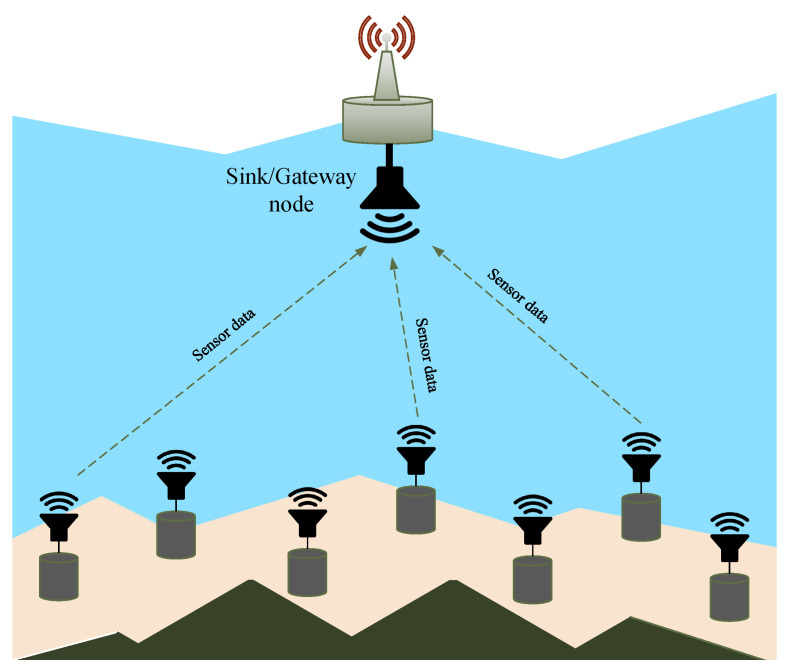
Considered UASN model.

**Figure 2:**
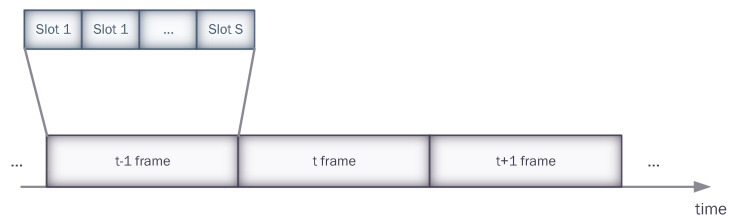
Slotted time-frame structure.

We consider a network of *N* underwater sensor nodes that transmit data to a common sink node utilizing acoustic communication (Figure 1). This type of network topology is common for a variety of practical applications, such as long-term environmental monitoring, seismic monitoring in oil reservoirs, and the Internet of Underwater Things. To avoid collisions at the sink node, the nodes of a MAC protocol are arranged in a time-slotted manner. Time is divided into frames, each consisting of *S* time slots, as illustrated in Figure 2. Each node is allowed to transmit sensed data once per frame. To transmit a data packet, a node needs to select one of the *S* available slots in the subsequent frame. Once a packet is received, a sink uses a separate downlink channel to broadcast the acknowledgment (ACK) of reception. The system model considered in this study is identical to that used in [3].

The proposed MAC protocol relies on reinforcement learning to alleviate the collision problem and achieve high channel utilization. In the considered reinforcement learning framework, sensor nodes are regarded as the learning agents that choose transmission strategies in each frame based on the feedback received from the sink node for their past actions. In the rest of this section, we first review the basic principles of reinforcement learning and Q-learning, which are the theoretical basis for the proposed DR-ALOHA-Q protocol. Then, we describe how those learning concepts are incorporated into the protocol design.

### 3.1. Reinforcement Learning Technique

Reinforcement learning (RL) is a type of machine learning that involves training an agent to make decisions that maximize a reward signal over a time horizon. The agent interacts with the environment in discrete time steps by performing actions and receives feedback in the form of a reward or penalty for those actions. Based on the observed feedback, the agent adjusts its behavior in an attempt to improve its decision-making skills over time through a trial-and-error process.

**Figure 3:**
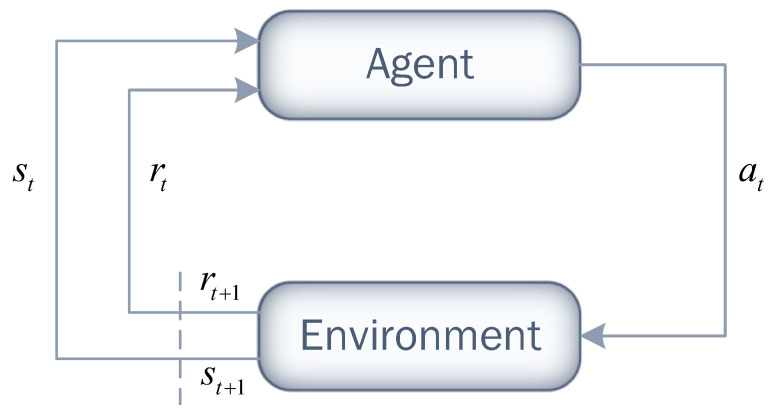
Reinforcement learning process.

Figure 3 illustrates the procedure followed by the RL agent in each time slot (*t*). The agent observes the current state of the environment ($s_{t}$) and selects an action ($a_{t}$) based on its current policy (π). In the context of RL, a policy (π) is a function that maps state–action pair $\left(s_{t},a_{t}\right)$ to the probability ($\pi (s_{t},a_{t})$) of selecting action $a_{t}$ in state $s_{t}$. After taking action $a_{t}$, it receives a direct reward ($r_{t}$). The agent uses this reward to evaluate its performance, and the environment moves to the next state ($s_{t+1}$). This process repeats at each time step.

Q-learning is one of the most popular model-free RL algorithms. In Q-learning, the agent aims to find a policy that maximizes the cumulative discounted reward, which is calculated by summing the rewards received at each time slot and discounting them by a factor between 0 and 1. This is achieved using a lookup Q table, which is a matrix representing the agent’s knowledge of the environment. The Q table comprises a row for each possible state of the environment and a column for each possible action the agent can take in that state. A matrix value ($Q(s_{t},a_{t})$) should reflect the expected reward that can be received by taking action $a_{t}$ in state $s_{t}$ and following the optimal policy from that point on. The optimal policy is greedy with respect to Q values of state–action pairs. The Q table is initially filled with random values, and the agent updates these values as it interacts with the environment according to the Bellman equation:

(1) $$Q\left(s_{t},a_{t}\right)\leftarrow \left(1-\alpha \right)Q\left(s_{t},a_{t}\right)+\alpha \left(r_{t+1}+\gamma \max_{a^{,}}Q\left(s_{t+1},a^{,}\right)\right)$$

where α∈(0,1] is the learning rate of the algorithm, and γ∈(0,1] is the discount factor.

### 3.2. DR-ALOHA-Q MAC Protocol

In the DR-ALOHA-Q protocol, sensor nodes use a Q-learning scheme to select a time slot in the frame to transmit a data packet. Additionally, nodes choose a time offset to delay packet transmission within the time slot. The protocol provides support for asynchronous network operation. The lack of reliance on time synchronization is considered an important advantage in UASNs, since GPS is not available underwater, and network-wide synchronization is costly and complex to implement. In contrast, terrestrial radio networks (TRNs) can be relatively easily synchronized based on the global time reference, reducing the likelihood of collisions in contention-based MAC schemes by decreasing the duration of the vulnerable period. The slotted ALOHA protocol approximately doubles the throughput of pure ALOHA in TRNs, since collisions only occur when synchronized transmissions are made in the same time slot [32].

**Figure 4:**
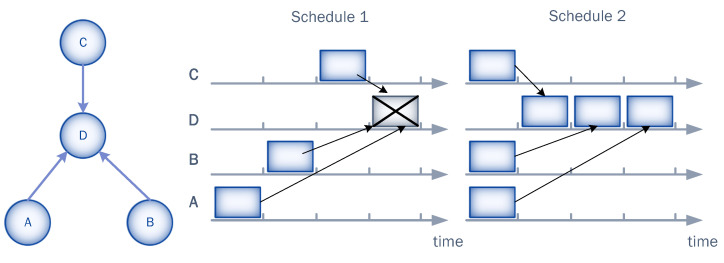
Space-time uncertainty in UASNs.

In UASNs, due to space-time uncertainty, the probability of collision depends on both transmission times and locations of sensor nodes, as illustrated in Figure 4 For instance, nodes A, B, and C can transmit at different time slots (Schedule 1) but cause collisions at D because of their different propagation delays. Alternatively, the nodes can transmit in the same time slot (Schedule 2) and avoid collisions at D if the difference between their propagation delays is large enough, i.e., larger than the product of the packet transmission time and the acoustic propagation speed in the water. Although this two-dimensional uncertainty can technically occur in any type of communication, it is only relevant when the propagation delay is significantly larger than the transmission delay, as in the case of UASNs. Therefore, to reduce the probability of collision in UASNs, it is necessary to eliminate the uncertainty in both dimensions. Network-wide synchronization can eliminate transmission time uncertainty, but eliminating space uncertainty requires the use of a slot size that can accommodate packet transmission time ($T_{p}$), maximum propagation delay in the network($\tau_{prop}^{max}$), and a guard interval ($T_{g}$). Considering that the physical properties of the acoustic underwater channel cause long propagation delays, dimensioning the time slot in this way significantly reduces channel utilization and increases end-to-end delay. Therefore, we decided to reduce the slot size to $T_{s}=T_{p}+T_{g}$ and to use a frame structure with S=N time slots. The guard interval is designed to efficiently handle variations in propagation delays that occur as a result of node mobility and changes in the water temperature, pressure, and salinity, as well as to enable fast convergence of the RL algorithm. The reduced slot size and asynchronous node operation result in unresolved time and space uncertainty and, in turn, an increased vulnerability (collision) period. However, DR-ALOHA-Q efficiently avoids collisions through Q-learning, as described below.

#### 3.2.1. A Model for Transmitting Packets

We now design a Q-learning model for DR-ALOHA-Q. Specifically, the definitions of system state, action space, and reward function in the DR-ALOHA-Q algorithm are given as follows.

**System state**. In the proposed protocol, the state is defined as the receiver of a data packet. Because the considered network model has only one node that acts as a data packet receiver, i.e., sink, the applied Q-learning method is a single-state Q-learning method. Several recent studies [3,7,26] adopted single-state Q-learning in the design of MAC protocols for underwater and terrestrial networks due to its low computational complexity. The reason for this low computational complexity lies in the fact that is difficult to define meaningful and non-misleading states for the MAC problem. The convergence time of multistate learning algorithms also might be unacceptably large for practical UASN applications. Therefore, we anticipate that single-state Q-learning is the most appropriate RL formulation, although the optimality of the solution is most likely sacrificed.

**Action space**. Actions concern transmission strategy decisions, i.e., joint selection of a time slot in the frame and a time offset (delay) for packet transmission within the time slot. We express the time-offset options as a multiple of δ time units. Let the maximum allowable time offset for packet transmission in each time slot be Kδ. Then, for each underwater node (*i*), the set of available actions is:

(2) $$A_{t}=\left\{a_{t}=\langle j,k\rangle |j\in S,k\in \left[0,1,2,\cdots ,K\right]\right\}$$

where 〈j,k〉 is the action of transmitting in *j*-th slot of the subsequent frame with a delay of $k\dot{\delta }$ time units from the start of the slot.

**Reward function**. After a sensor node performs an action, it evaluates the effectiveness of the action based on the reward ($r_{t+1}$) it receives. The goal of the Q-learning agent is to maximize the total network throughput. Therefore, the reward value for a given action ($a_{t}$) is based on the packet reception result of the sink node. Specifically, the sensor node waits for the acknowledgment (ACK) from the sink node and determines the observation as $o_{t}=\langle Success\rangle$ or 〈Collision〉. The deadline for ACK reception is designed to be larger than $2\tau_{prop}^{max}$. If the ACK is received before the deadline expires, the performed action is paired with the observation 〈Success〉; otherwise, 〈Collision〉 is implied. The reward function is defined as:

(3) $$r_{t+1}=\left\{\begin{array}{cc} 1, & \mathrm{if}\;o_{t}=\langle Success\rangle .\\ 0, & \mathrm{if}\;o_{t}=\langle Collision\rangle . \end{array}\right.$$

#### 3.2.2. Learning to Avoid Collisions

To determine the optimal transmission strategy, every time a node needs to transmit a data packet, it checks the Q values of all possible actions. The Q values are maintained in the form of a one-dimensional Q table with a number of elements equal to S·K, which corresponds to the size of the action space. The table is initialized with random values between 0 and 1 so that all nodes start learning with random action choices. At the start of each time frame, every node selects a time slot and a time offset for the slot as indicated by the action with the largest Q value. The Q-table is updated according to the observed transmission result (i.e., the ACK signal) as follows:

(4) $$Q_{t+1}\left(a\right)=Q_{t}\left(a\right)+\alpha \left(r_{t+1}-Q_{t}\left(a\right)\right)$$

**Figure 5:**
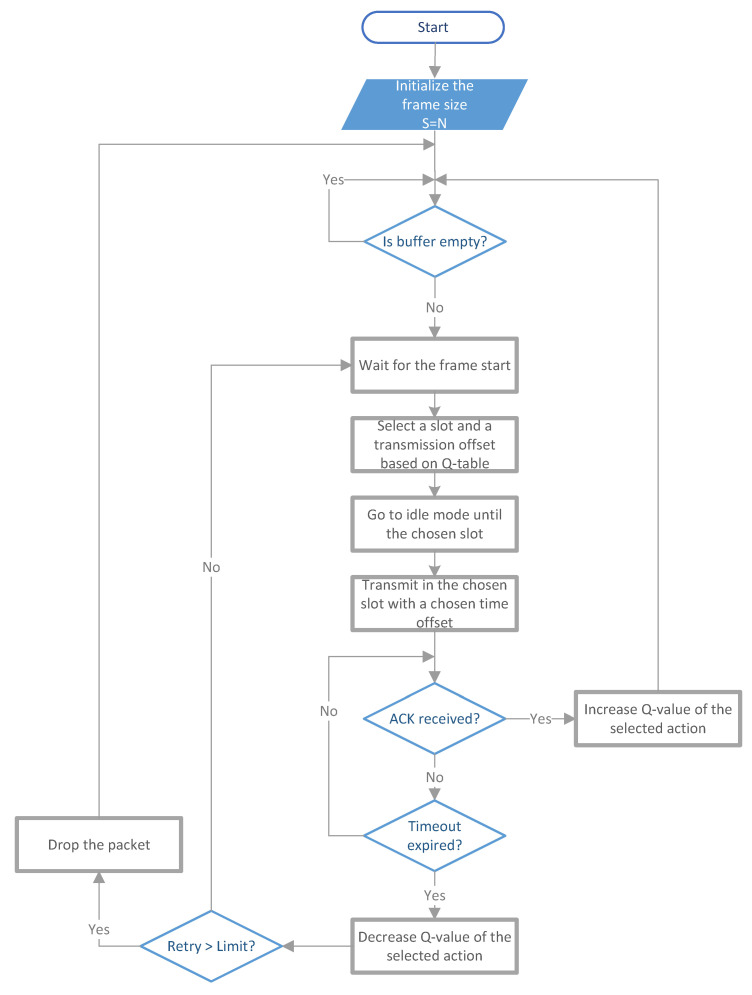
Flow chart of the DR-ALOHA-Q protocol.

In the above equation, *t* denotes the start time of the frame in which action *a* is taken, while t+1 is the time when the observation of the transmission result is determined, i.e., the moment when the ACK is received or the deadline for receiving the ACK expires. The Q-value updating rule differs from (1) because in single-state Q-learning, there is no new state and, thus, no concept of future reward. The protocol operation is presented visually through a flow chart in Figure 5.

It should be noted that the proposed Q-learning model violates the implicit assumption of conventional RL that the agent takes a new action only after it receives the corresponding feedback for the action performed in the previous time step [3]. Because of the reduced time-slot size, ACKs for successful transmission may not arrive during the same time frame. This is particularly true for sensor nodes far away from the sink node, the propagation delay of which could be many times larger than the frame size. While the reduced slot size entails higher theoretical channel utilization, delayed rewards slow down the learning process. We investigate this tradeoff in Section 4.

In the proposed DR-ALOHA-Q protocol, network nodes act as independent learning agents that share the same goal: maximizing the network throughput. Independent learning makes the protocol scalable because the size of Q tables is not affected by the number of nodes. However, it also makes the environment unpredictable from the local perspective of an agent. To speed up the convergence time of the distributed learning, we apply a variant of multiagent RL called Hysteretic Q-learning [8]. In the multiagent environment, even if an agent chooses a good action, it may suffer a penalty (i.e., reward 0) as a result of actions taken by the other nodes. To address this issue, in compliance with hysteretic Q-learning principles, we use two different learning rates, α and β, in the Q-table update rule, as follows:

(5) $$\mu =r_{t+1}-Q_{t}\left(a\right)$$

(6) $$Q_{t+1}\left(a\right)\leftarrow \left\{\begin{array}{cc} Q_{t}\left(a\right)+\alpha \cdot \mu , & \mathrm{if}\;\mu \geq 0\\ Q_{t}\left(a\right)+\beta \cdot \mu , & \mathrm{else} \end{array}\right.$$

These learning rates are determined based on the sign of the parameter μ, which indicates whether the actions taken were beneficial or detrimental to achieving the desired behavior of the system. Learning rate α is used for increasing Q values when the actions taken were beneficial, while β is used for decreasing Q values when the actions taken were detrimental. In order to give less weight to penalties, β is set to a value lower than α. Therefore, an agent places less emphasis on a penalty that was incurred for actions that were previously awarded.

## 4. Simulation Results

In this section, we assess the effectiveness of DR-ALOHA-Q through simulations. The simulations were conducted using our self-developed event-driven simulator, which was implemented in Python programming language We compare the performance of the proposed protocol with that of UW-ALOHA-Q, DOTS, and CS-ALOHA in the UASN scenario with periodic data gathering under saturated traffic conditions. The simulation analysis is conducted for random network typologies with 10 and 25 sensor nodes. The network radius is varied from 100 m to 1500 m. Unless otherwise specified, simulation parameters from Table 1 are used. The data packet size and ACK size in bits were determined based on prior research [3]. To suit the practical settings of underwater environments, a data rate of 13,900 bps was assumed by referring to an underwater modem that is currently available on the market [33]. The protocol performance is evaluated in both static and mobile network setups. All nodes are considered to be within interfering range of each other. For the proposed protocol and UW-ALOHA-Q, the simulation duration is set to 3000 time frames. DOTS and CS-ALOHA were evaluated for the same amount of time as the proposed protocol.

### 4.1. Benchmark Protocols

DR-ALOHA-Q is compared to three previously proposed underwater MAC techniques, each presenting a different and paradigmatic approach to collision avoidance. UW-ALOHA-Q is a reinforcement-learning-based MAC protocol designed for underwater communication. It utilizes Q-learning to determine the optimal time slot for transmitting data packets in each time frame. Compared to the ALOHA-Q protocol, which is used in terrestrial networks, UW-ALOHA-Q introduces three enhancements to address the unique challenges of the underwater environment: refined frame length, asynchronous operation, and uniform random backoff. To improve channel utilization, UW-ALOHA-Q uses a refined frame size with *S* time slots, where *S* is computed based on the index ratio (*B*), defined as:

(7) $$B=\frac{S\cdot (2\cdot \tau_{prop}^{max}+T_{p})}{N\cdot T_{p}}$$

In particular, UW-ALOHA-Q proposes to choose the number of slots in order to maximize channel utilization under the condition that the index B is equal to or greater than 1.5, assuming that the number of nodes in a network (N) and the network radius (R) are known.

The DOTS protocol uses passively observed information about the neighboring transmissions to achieve both temporal and spatial reuse in UASNs. Each node builds a delay map, which includes information about the source and destination of the observed packets, the time at which packets were sent (timestamps), and the estimated propagation delay between the packet source and the destination. The timestamps included in the packet header are used to calculate the propagation delay to the packet sender. However, this requires clock synchronization across the network, which is difficult to achieve in UASNs. Based on the overheard transmissions, each node can estimate the time at which a response to the observed packet will arrive back to the sender. When a node needs to transmit a frame, it employs a decision-making algorithm based on the delay map database to determine whether or not to initiate the transmission. If no conflicts arise, the node starts transmitting. If conflicts are detected in the delay map, the node delays its transmission for a random period of time (backoff period) before attempting transmission again.

CS-ALOHA is a modified version of ALOHA that is suitable for use in underwater settings. In this approach, nodes transmit their data without engaging in the RTS/CTS handshaking process, as long as the channel is not currently in use. Additionally, the system includes an acknowledgment mechanism to confirm successful transmissions. For both CS-ALOHA and DOTS, we set the maximum backoff period to $10\tau_{prop}^{max}$, following the approach proposed in [16].

### 4.2. Investigated Metrics

The protocol performance is evaluated through the investigation of the channel utilization (*U*) and protocol convergence time ($C_{t}$). Channel utilization is defined as the fraction of the total simulation time during which the sink node successfully receives data traffic:

(8) $$U=\frac{D\cdot T_{p}}{Simulation\;duration}$$

where *D* is the total number of successfully received data packets.

The protocol convergence time ($C_{t}$) is evaluated as the time required to find the optimal transmission strategy for a certain network size. Since UW-ALOHA-Q and DR-ALOHA-Q use different slot sizes, we normally express the convergence time in seconds. However, we also present results in terms of the number of time frames required for the protocol to converge, since this better indicates the energy efficiency of the protocol. The performance metrics are measured in a consistent manner as an average of 20 simulation runs.

### 4.3. Simulation of Static UASN

This scenario pertains to UASNs that comprise nodes that are either moored or anchored. The unintentional movements of sensor nodes are neglected.

**Figure 6:**
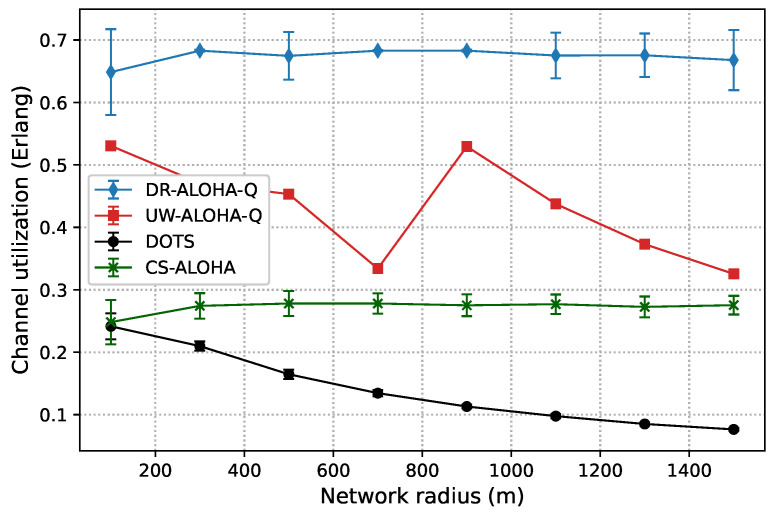
Channel utilization for a static UASN comprising 10 nodes.

**Figure 7:**
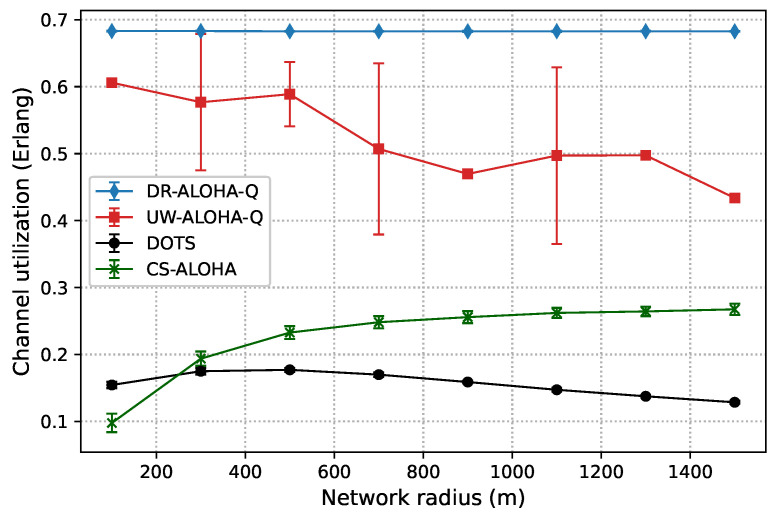
Channel utilization for a static UASN comprising 25 nodes.

Figure 6 and Figure 7 show the channel utilization achieved by the analyzed protocols in networks with 10 and 25 nodes randomly deployed within a circle area, respectively. The results clearly indicate that DR-ALOHA-Q achieves the highest channel utilization and relatively steady-state performance. The DOTS protocol exhibits the worst behavior. The throughput of DOTS decreases in more sparsely deployed networks. This is a general feature of handshaking-based protocols. As the distance between nodes increases, the overall time required to reserve the channel and transmit data also increases, which reduces the network throughput. In sparsely deployed networks, DOTS nodes are less likely to detect active transmissions in the network and therefore fail to efficiently exploit temporal reuse. CS-ALOHA outperforms DOTS in almost all the analyzed network configurations. In the UASN scenario with 25 nodes, CS-ALOHA exhibits very low channel utilization for short network radii because the nodes often overhear transmissions from other nodes but are unable to identify collision-free transmission opportunities, leading to unnecessary backoff periods. As a result, channel utilization is significantly reduced, and the overall performance of the network is adversely affected. Moreover, it is important to note that the throughput gains of CS-ALOHA, compared to DOTS, come at the cost of lower energy efficiency and fairness due to the higher number of transmission attempts (as well as collisions) and the lack of a fairness control mechanism [9]. The throughput performance of CS-ALOHA is generally inferior to that of learning-based protocols such as UW-ALOHA-Q and DR-ALOHA-Q. Both UW-ALOHA-Q and DR-ALOHA-Q tend to imitate TDMA scheduling. Therefore, the fairness of these protocols is implied.

The channel utilization of UW-ALOHA-Q exhibits a zig-zag pattern due to its utilization of large time slots and the selection of the optimal number of slots in a frame based on (7). This leads to varying amounts of idle time in a frame depending on the maximum propagation delay or network radius. For instance, in a network with 25 nodes, the optimal number of slots varies for different network sizes. A network with a radius of 100 m requires 14 slots for optimal channel utilization, while a network with a radius of 900 m only needs 3 slots. This leads to maximum theoretical utilization of 0.6058 Erlangs and 0.469 Erlangs for the former and latter cases, respectively. This effect is discussed in [7] and termed as “the effect of a slot”. On the contrary, DR-ALOHA-Q has a maximum theoretical channel utilization that does not vary with the network radius, as it only depends on the slot size. The number of slots is always equal to the number of network nodes, which eliminates the zig-zag pattern in channel utilization. We set the default slot size to 110 ms, i.e., ∼$1.46T_{p}$. This allows the slot to accommodate packet transmission time and a guard interval. The size of the guard interval is determined empirically based on a simulation study that considers the tradeoff between the protocol throughput and convergence properties. With this setting, the maximum theoretical channel utilization is approximately 0.6828 Erlangs.

It is interesting to note that DR-ALOHA-Q achieved slightly worse results in the scenario with 10 nodes than in the scenario with 25 nodes. This could be due to the fact that nodes transmit more often. Although the majority of nodes can promptly identify a collision-free transmission approach, some nodes may fail to do so because the Q values of other nodes’ decisions rise steeply due to the rarity of collisions in comparison to the number of successful transmissions. The problem could be alleviated by using a more sophisticated algorithm to adjust the learning rate or a more granular reward mechanism, which would favor the nodes experiencing a large number of consecutive collisions. As the primary aim of this paper is to demonstrate improved performance compared to UW-ALOHA-Q, which can be accomplished with larger slot sizes, as demonstrated later, we leave further improvements of the learning algorithm for our future work.

**Figure 8:**
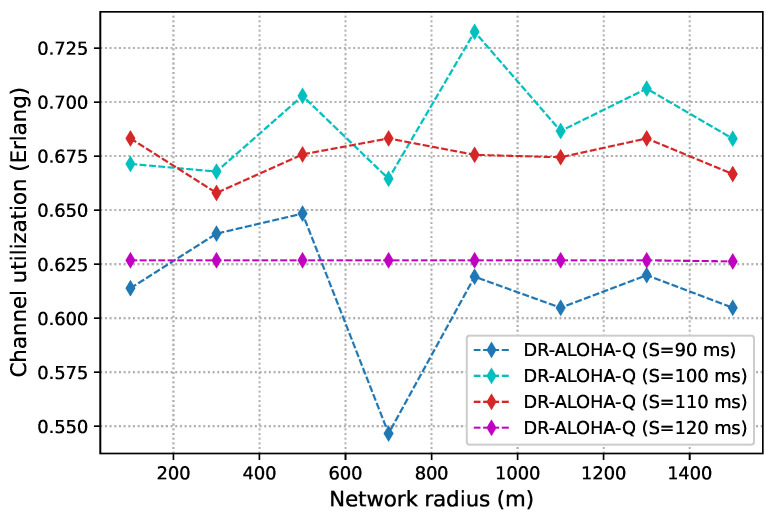
DR-ALOHA-Q channel utilization as a function of slot size in a UASN with 10 nodes.

**Figure 9:**
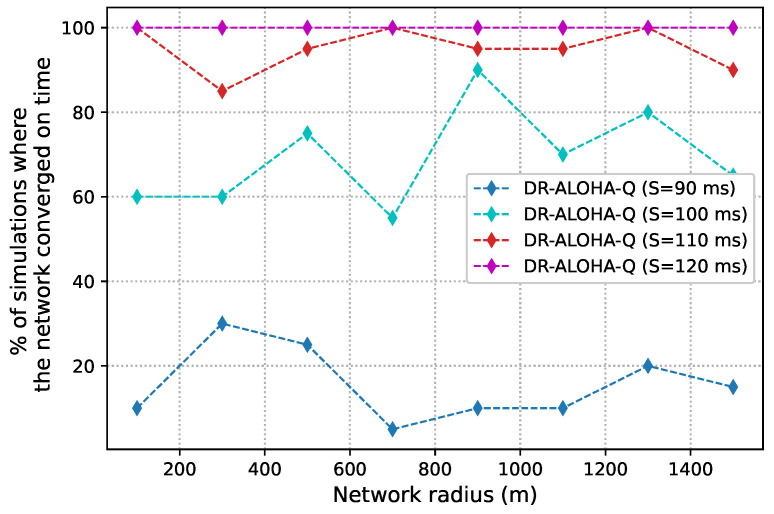
Convergence rate of DR-ALOHA-Q in a UASN with 10 nodes.

**Figure 10:**
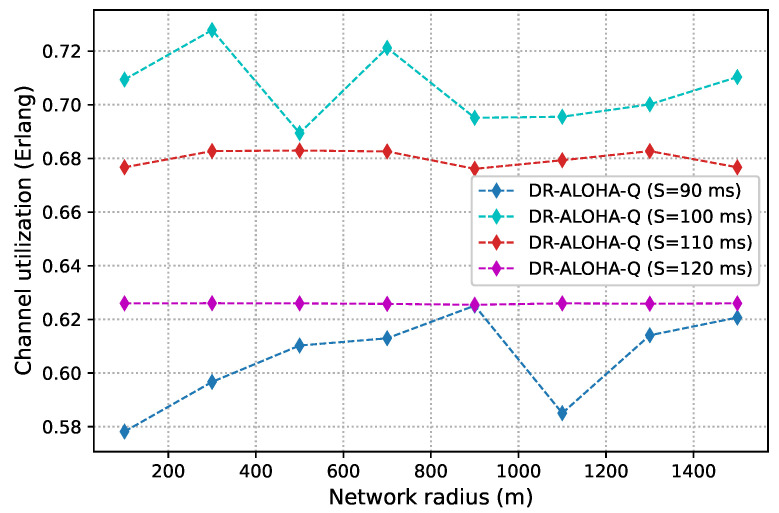
DR-ALOHA-Q channel utilization as a function of slot size in a UASN with 25 nodes.

**Figure 11:**
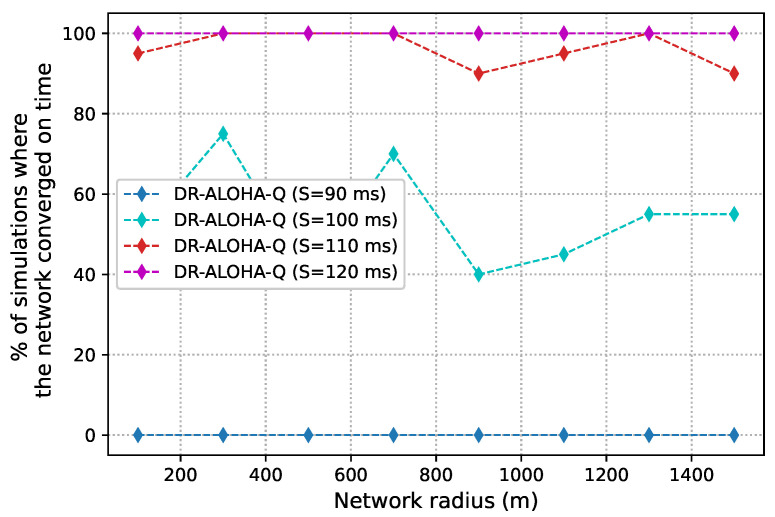
Convergence rate of DR-ALOHA-Q in a UASN with 25 nodes.

As discussed before, slot size is a crucial parameter that determines the achievable channel utilization of DR-ALOHA-Q. Moreover, it impacts the end-to-end communication delay. If the slot size is too large, the channel utilization will be low. Conversely, if the slot size (i.e., guard interval) is too small, nodes will fail to find a collision-free transmission strategy. Figure 8 presents simulation results in terms of channel utilization for a 10-node network achieved using slot sizes of 90, 100, 110, and 120 ms, which corresponds to approximately $1.2T_{p},1.3T_{p},1.5T_{p}$, and $1.6T_{p}$, respectively. Figure 9 shows the percentage of simulations in which the network converges before 3000 iterations. Convergence is considered to have occurred when all nodes transmit packets using the same action (slot and offset) for 20 consecutive frames. For a network with 25 nodes, similar results are shown in Figure 10 and Figure 11. The results clearly indicate that the learning algorithm used by DR-ALOHA-Q cannot converge in a reasonable time when the slot size is $1.2T_{p}$ and $1.3T_{p}$. When the slot size is equal to approximately $1.5T_{p}$, the protocol converges to the optimal scheduling solution in most of the cases. In both scenarios, i.e., for networks comprising 10 and 25 nodes, convergence can be guaranteed with a slot size of $1.6T_{p}$. This slot size configuration corresponds to the maximum achievable channel utilization of 0.63 Erlangs, which is still considerably better than that achievable with UW-ALOHA-Q.

Table 2 and Table 3 present a comparison of the convergence properties of the proposed protocol and UW-ALOHA-Q for UASNs with 10 and 25 nodes, respectively. In each case, 20 simulations were conducted for different network radii. Since the protocols do not use the same frame size, the convergence time is expressed in seconds. In order to provide a comprehensive evaluation, we report the average result of all simulation runs where the protocols converged, as well as the percentage of simulations where the protocols achieved convergence. Table 3 shows that UW-ALOHA-Q successfully converges in less than 3000 frames in the UASN scenario with 10 nodes. This is not solely due to reduced contention among the nodes but also due to significant idle time in a frame as a result of the calculation (7). Namely, UW-ALOHA-Q has a maximum theoretical channel utilization of less than 0.55 in all of the examined network configurations. When the number of network nodes is 25, UW-ALOHA-Q fails to converge in up to 35% of simulations for certain network configurations, as shown in Table 3. In this situation, the duration of idle time within the frame is decreased due to the implementation of (7) as a frame dimensioning mechanism. For instance, in a network with a radius of 700 m, the maximum theoretical channel utilization of UW-ALOHA-Q is approximately 0.59. Although this is much lower than the channel utilization offered by DR-ALOHA-Q, UW-ALOHA-Q struggles to converge in 35% of simulation runs.

In the scenario with 25 nodes, DR-ALOHA-Q converges in 100% of situations for both S=110 ms and S=120 ms Therefore, the convergence time is around 250 s in the worst case, which is significantly lower than the convergence time of UW-ALOHA-Q in most of the cases. The only scenario in which UW-ALOHA-Q achieves faster convergence is that in which the network radius is 900 m. However, in this scenario, the maximum channel utilization offered by UW-ALOHA-Q is considerably lower than that of the proposed protocol, as shown in Figure 6. In a network with 10 randomly deployed nodes, the convergence rate of DR-ALOHA-Q for S=110 ms is slightly worse due to the occasional oscillation in learning explained before. However, relative to UW-ALOHA-Q, throughput gain is even more pronounced. With S=120 ms, DR-ALOHA-Q achieves stable channel utilization of 0.626 Erlangs, while its convergence time is negligibly slower (up to 15 s) in the worst-case scenario and significantly faster (over 7 times) in some cases. It is important to note that UW-ALOHA-Q achieves faster convergence only when it uses a frame structure with a long idle time, which significantly lowers channel utilization compared to DR-ALOHA-Q.

**Figure 12:**
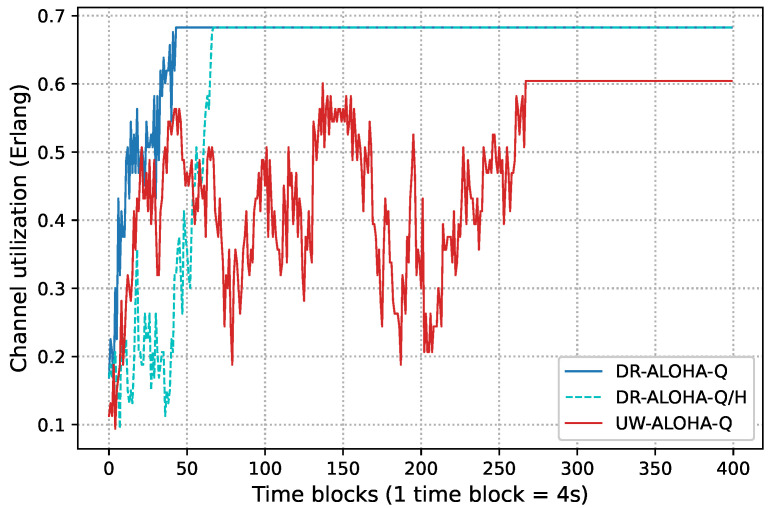
Real-time channel utilization in a 500 m UASN for the UW-ALOHA-Q and the proposed protocol with DR-ALOHA-Q and without hysteretic Learning (DR-ALOHA-Q/H).

Figure 12 shows the channel utilization as a function of time for DR-ALOHA-Q with/without hysteretic Learning (HL) and UW-ALOHA-Q. The results refer to a network topology comprising 25 nodes randomly deployed within a circular area of a 500 m radius. Figure 12 shows that both DR-ALOHA-Q and UW-ALOHA-Q nodes successfully learned an optimal transmission strategy. However, it can also be observed that DR-ALOHA-Q converged faster in the learning process and that HL contributed to decreased convergence time. Therefore, DR-ALOHA-Q achieved higher channel utilization than UW-ALOHA-Q.

**Figure 13:**
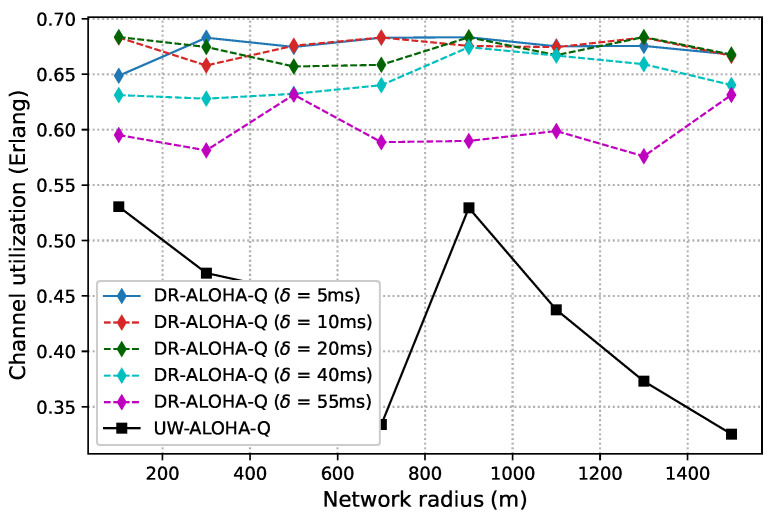
DR-ALOHA-Q channel utilization as a function of δ in a UASN with 10 nodes.

**Figure 14:**
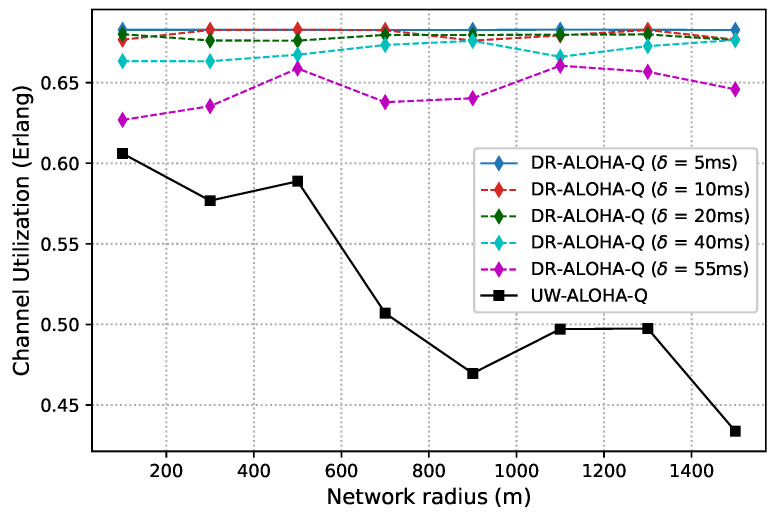
DR-ALOHA-Q channel utilization as a function of δ in a UASN with 25 nodes.

One of the key DR-ALOHA-Q features is the ability to delay a packet transmission with a time slot to leverage space-time uncertainty and enhance throughput. Thus, we analyzed the impact of the time-offset step (δ) on the protocol performance. In particular, the protocol performance is evaluated for δ∈[5,10,20,40,55] ms, and the obtained results in terms of channel utilization are presented in Figure 13 for the UASN scenario with 10 nodes and in Figure 14 for the UASN scenario with 25 nodes. The results in terms of convergence properties are reported for a network radius of 500 m in Table 4 and Table 5. The results show that the performance deteriorates with an increase in δ, but decent throughput performance can be achieved even with relatively large time-offset steps. A small value for δ can help the protocol converge to the optimal strategy, but if the value is too small, the convergence process might be slowed down significantly due to an increased action space of the learning algorithm. On the other hand, when offset steps are less granular, the protocol either converges rapidly (because action space is relatively small) or fails to converge at all. In networks with anchored/moored sensor nodes, achieving network convergence is considered the primary objective. After convergence, a globally optimal packet transmission schedule is formed, and the nodes can stop the learning algorithm and just repeat the learned schedule as long as network convergence is maintained. In mobile networks, convergence does not apply; therefore, the focus is on retaining high channel utilization through effective adaptation of transmission timings in response to changing conditions. The value of the δ parameter should be chosen by carefully considering a tradeoff between channel utilization and the likelihood of convergence for a given network scenario.

### 4.4. Simulation of a Free-Floating UASN

In addition to static typologies, we evaluated the proposed protocol in a mobile UASN with free-floating sensor nodes. In particular, *N* nodes are randomly deployed in a circle area with a 500 m radius and moved according to the meandering current mobility (MCM) model [34] for 30 min. The node movements are limited to the circle region with a radius of 1000 m around the fixed sink node. In this way, all deployed nodes remain within an interference range of each other during the whole simulation and experience a high level of channel contention. The MCM mobility model operates under the assumption that sensor nodes are not independent in their movement. Instead, they are advected by the same velocity field, which, in turn, is affected by meandering subsurface currents and vortices, influencing the nodes’ path vectors. To maintain consistency with [9], we restricted node speed to 0.3 m/s in our simulations by parameterizing the MCM model accordingly.

**Figure 15:**
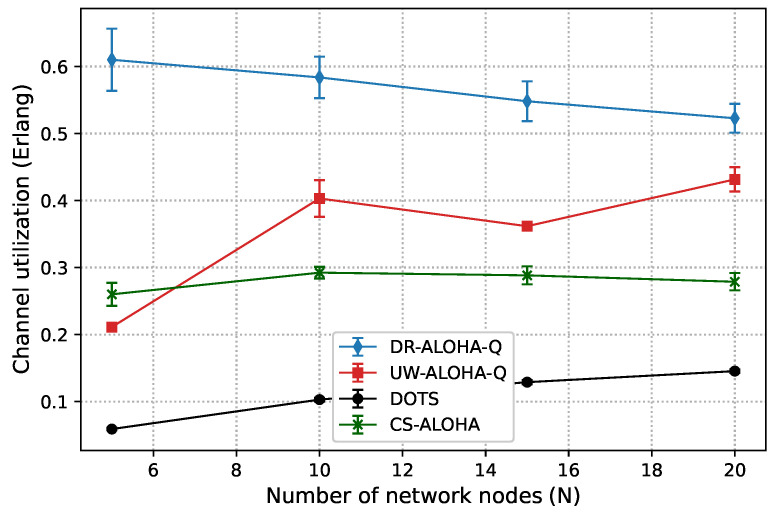
Channel utilization in a free-floating UASN.

The effect of MCM mobility on channel utilization of the proposed protocol and UW-ALOHA-Q, DOTS, and CS-ALOHA protocols is examined in Figure 15. The protocols are evaluated in scenarios with different numbers of sensor nodes, specifically N∈[5,10,15,20]. The presented results are averaged over 20 simulation runs, with error bars representing the standard deviation of the results. All protocols are tested using the same set of parameters as in the static UASN scenario, with the exception of the DOTS protocol. To mitigate invalid transmission scheduling caused by node mobility, a 20 ms-long guard interval is utilized for DOTS, as proposed in [9]. The obtained results clearly demonstrate the superior performance of DR-ALOHA-Q over UW-ALOHA-Q, DOTS, and CS-ALOHA in the scenario with MCM mobility. By incorporating the learning-based adaptive MAC approach, DR-ALOHA-Q effectively leverages time-space uncertainty, resulting in significant improvements in channel utilization and throughput. In general, the efficiency of learning-based approaches largely depends on the frequency and magnitude of environmental changes. When the rate of environmental changes is low, learning-based protocols are more likely to deliver superior performance outcomes. In contrast, in situations in which environmental changes occur rapidly and unpredictably, the effectiveness of these approaches may be diminished. Nevertheless, in the considered mobile UASN scenario, DR-ALOHA-Q adapts to node movements sufficiently rapidly, resulting in a notably higher channel utilization compared to the other protocols.

Compared to a static network scenario, node mobility considerably deteriorates the performance of UW-ALOHA-Q In the given scenario, the maximum theoretical channel utilization achievable with UW-ALOHA-Q protocol is approximately 0.21, 0.48, 0.37, and 0.5 Erlangs for N∈[5,10,15,and20], respectively. Consequently, the idle time at the sink node constitutes 79%, 52%, 63%, and 50% of the frame duration for these network sizes when the optimal transmission schedule is established. This idle time serves as a guard interval to account for variations in propagation delay that arise due to node mobility, helping to prevent collisions and ensure reliable data transmission between the nodes and the sink. However, the guard or idle intervals are not evenly distributed within a frame. Thus, although these intervals may be relatively large, they cannot always absorb the node movements. In some cases, adjustments to the transmission strategy are required in order to maintain optimal throughput performance. As shown in Figure 15, in the scenarios with N=10 and N=20 nodes, the MCM mobility model deteriorates UW-ALOHA-Q’s channel utilization by 20% and 16%, respectively, compared to the maximum theoretical values. In the scenarios with N=5 and N=15 nodes, there was no performance degradation due to node mobility, but the achieved channel utilization is generally low due to previously elaborated limitations of the frame dimensioning formula.

In comparison to UW-ALOHA-Q, DR-ALOHA-Q employs smaller idle intervals at the sink, which account for just 38% of the frame duration. This entails that DR-ALOHA-Q nodes need to adjust transmission strategies more frequently. Notably, as the number of nodes (*N*) increases, the performance degradation of DR-ALOHA-Q compared to a static network scenario becomes more pronounced. However, DR-ALOHA-Q still outperforms UW-ALOHA-Q by a considerable margin of 23% in the worst-case scenario (N=20) and by nearly 200% in the best-case scenario (N=5). This superior performance can be attributed to two main factors. First, the maximum theoretical channel utilization of DR-ALOHA-Q is much higher (0.683 Erlangs). Secondly, DR-ALOHA-Q is able to learn good policies more rapidly without relying on the unstable and inefficient uniform random backoff mechanism. In the considered fully connected network scenarios, mobility did not cause any considerable changes in throughput for DOTS and CS-ALOHA.

**Figure 16:**
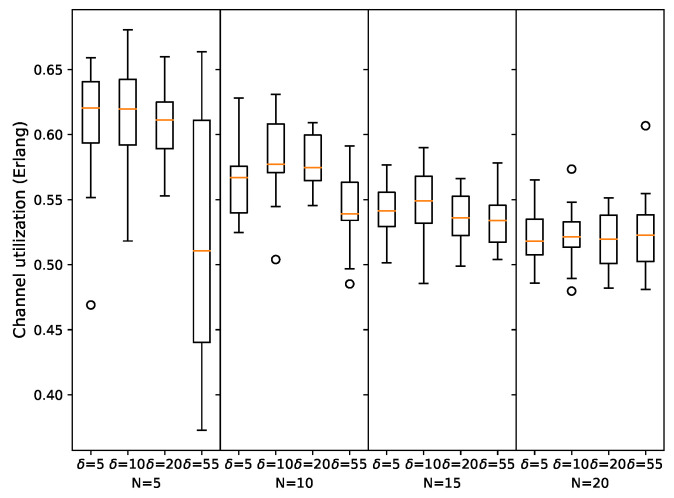
DR-ALOHA-Q channel utilization as a function of δ in a free-floating UASN scenario.

As depicted in Figure 16, we also analyzed the impact of the time-offset step (δ) on the protocol performance in a free-floating network. Our results indicate that even for δ=55 ms, which corresponds to half the slot size, DR-ALOHA-Q achieves a respectable channel utilization. However, when the number of nodes is small (e.g., N=5), the benefits of using small offset steps are more evident. In larger networks, finding an efficient transmission strategy becomes a more complex task. Therefore, using a small δ does not necessarily improve channel utilization, as the environment changes faster than the learning algorithm can converge.

## 5. Conclusions

In this paper, we presented DR-ALOHA-Q, a delayed-reward ALOHA-Q for UASNs. DR-ALOHA-Q utilizes Q-learning algorithms to determine optimal time slots and time offsets within a slot for data transmissions by sensor nodes. In this way, the protocol leverages large propagation delays in underwater acoustic communication to maximize channel utilization. Therefore, the protocol operates without any prior knowledge of link propagation delays and does not require time synchronization across the network. Each node learns the optimal transmission strategy independently, solely based on the delayed feedback from the sink node. We evaluated the performance of DR-ALOHA-Q by means of simulations in static and free-floating UASNs of different sizes under saturated traffic load. The performance of the protocol was compared to that of the state-of-the-art reinforcement-learning-based MAC protocol available in the literature, namely UW-ALOHA-Q, as well as two other popular MAC approaches, DOTS and CS-ALOHA. The results show that DR-ALOHA-Q not only achieves significant performance improvements in terms of channel utilization but also exhibits robustness against different propagation delays and demonstrates good adaptability to network mobility. In terms of channel utilization gains, DR-ALOHA-Q outperforms UW-ALOHA-Q, achieving gains ranging from 13% to 106% in a static network and from 23% to 200% in a mobile network scenario.dditionally, DR-ALOHA-Q effectively reduces end-to-end delay. The performance gains stem from a reduced time-slot size and a reinforcement learning framework that drives the protocol operations by letting each node to promptly adjust the transmission strategy in response to changing communication conditions.

In our future work, we aim to further improve the efficiency of the learning algorithm and extend the protocol to support the smooth integration of new nodes into an operating UASN.

## Figures and Tables

**Table 1:** Simulation parameters.

Parameter	Value
Data packet size	1044 bits
ACK packet size	20 bits
Data packet transmission time ($T_{p}$)	75.108 ms
ACK packet transmission time ($T_{a}$)	1.439
Slot duration	110 ms
Propagation speed	1500 m/s
Transmission rate	13,900 bps
Learning rate α	0.1
Learning rate β	0.01
Time-offset step δ	5 ms

**Table 2:** Convergence properties of DR-ALOHA-Q and UW-ALOHA-Q in the UASN scenario with 10 nodes.

Protocol Name	Network Radius in Meters	Convergence Time in Seconds	% of Simulations Where the Network Vonverges
DR-ALOHA-Q (S = 110 ms)	100	31.93	100%
DR-ALOHA-Q (S = 120 ms)	100	28.55	100%
UW-ALOHA-Q	100	25.93	100%
DR-ALOHA-Q (S = 110 ms)	300	31.26	85%
DR-ALOHA-Q (S = 120 ms)	300	26.18	100%
UW-ALOHA-Q	300	27.44	100%
DR-ALOHA-Q (S = 110 ms)	500	36.06	95%
DR-ALOHA-Q (S = 120 ms)	500	32.77	100%
UW-ALOHA-Q	500	23.69	100%
DR-ALOHA-Q (S = 110 ms)	700	40.89	100%
DR-ALOHA-Q (S = 120 ms)	700	34.55	100%
UW-ALOHA-Q	700	19.47	100%
DR-ALOHA-Q (S = 110 ms)	900	36.58	95%
DR-ALOHA-Q (S = 120 ms)	900	33.31	100%
UW-ALOHA-Q	900	245.41	100%
DR-ALOHA-Q (S = 110 ms)	1100	39.66	95%
DR-ALOHA-Q (S = 120 ms)	1100	34.66	100%
UW-ALOHA-Q	1100	168.02	100%
DR-ALOHA-Q (S = 110 ms)	1300	47.48	100%
DR-ALOHA-Q (S = 120 ms)	1300	39.58	100%
UW-ALOHA-Q	1300	120.12	100%
DR-ALOHA-Q (S = 110 ms)	1500	47.53	90%
DR-ALOHA-Q (S = 120 ms)	1500	41.69	100%
UW-ALOHA-Q	1500	92.54	100%

**Table 3:** Convergence properties of DR-ALOHA-Q and UW-ALOHA-Q in the UASN scenario with 25 nodes.

Protocol Name	Network Radius in Meters	Convergence Time in Seconds	% of Simulations Where the Network Vonverges
DR-ALOHA-Q (S = 110 ms)	100	199.48	100%
DR-ALOHA-Q (S = 120 ms)	100	135.86	100%
UW-ALOHA-Q	100	360.57	100%
DR-ALOHA-Q (S = 110 ms)	300	181.46	100%
DR-ALOHA-Q (S = 120 ms)	300	143.92	100%
UW-ALOHA-Q	300	2054.03	80%
DR-ALOHA-Q (S = 110 ms)	500	183.96	100%
DR-ALOHA-Q (S = 120 ms)	500	121.74	100%
UW-ALOHA-Q	500	3246.2	90%
DR-ALOHA-Q (S = 110 ms)	700	250.25	100%
DR-ALOHA-Q (S = 120 ms)	700	123.47	100%
UW-ALOHA-Q	700	1909.9	65%
DR-ALOHA-Q (S = 110 ms)	900	189	100%
DR-ALOHA-Q (S = 120 ms)	900	182.67	100%
UW-ALOHA-Q	900	177.97	100%
DR-ALOHA-Q (S = 110 ms)	1100	194.62	100%
DR-ALOHA-Q (S = 120 ms)	1100	132.26	100%
UW-ALOHA-Q	1100	1808.55	70%
DR-ALOHA-Q (S = 110 ms)	1300	208.66	100%
DR-ALOHA-Q (S = 120 ms)	1300	136.3	100%
UW-ALOHA-Q	1300	282.67	100%
DR-ALOHA-Q (S = 110 ms)	1500	244.46	100%
DR-ALOHA-Q (S = 120 ms)	1500	123.76	100%
UW-ALOHA-Q	1500	176.83	100%

**Table 4:** Convergence properties of DR-ALOHA-Q in function of δ in a static UASN with 10 nodes.

δ (ms)	Network Radius in Meters	Convergence Time in Frames	% of Simulations Where the Network Converges
5	500	50.58	95%
10	500	36.37	95%
20	500	40.23	85%
40	500	35	70%
55	500	26.29	70%

**Table 5:** Convergence properties of DR-ALOHA-Q as a function of δ in a static UASN with 25 nodes.

δ (ms)	Network Radius in Meters	Convergence Time in Frames	% of Simulations Where the Network Converges
5	500	59.2	100%
10	500	69.65	100%
20	500	55.72	90%
40	500	46.73	75%
55	500	44.77	65%

## Data Availability

Not applicable.

## References

[1] Jouhari M., Ibrahimi K., Tembine H., Ben-Othman J. (2019). Underwater Wireless Sensor Networks: A Survey on Enabling Technologies, Localization Protocols, and Internet of Underwater Things. IEEE Access.

[2] Sendra S., Lloret J., Jimenez J.M., Parra L. (2016). Underwater Acoustic Modems. IEEE Sens. J..

[3] Park S.H., Mitchell P.D., Grace D. (2019). Reinforcement Learning Based MAC Protocol (UW-ALOHA-Q) for Underwater Acoustic Sensor Networks. IEEE Access.

[4] Hsu C.C., Kuo M.S., Chou C.F., Lin K.C.J. (2013). The Elimination of Spatial-Temporal Uncertainty in Underwater Sensor Networks. IEEE/ACM Trans. Netw..

[5] Agiwal M., Roy A., Saxena N. (2016). Next Generation 5G Wireless Networks: A Comprehensive Survey. IEEE Commun. Surv. Tutorials.

[6] Gunn M., Koo S.G.M. (2009). A Comparative Study of Medium Access Control Protocols for Wireless Sensor Networks. Int. J. Commun. Netw. Syst. Sci..

[7] Park S.H., Mitchell P.D., Grace D. (2021). Reinforcement Learning Based MAC Protocol (UW-ALOHA-QM) for Mobile Underwater Acoustic Sensor Networks. IEEE Access.

[8] Matignon L., Laurent G.J., Le Fort-Piat N. Hysteretic Q-learning: An algorithm for Decentralized Reinforcement Learning in Cooperative Multi-Agent Teams. Proceedings of the 2007 IEEE/RSJ International Conference on Intelligent Robots and Systems.

[9] Noh Y., Wang P., Lee U., Torres D., Gerla M. DOTS: A propagation Delay-aware Opportunistic MAC protocol for underwater sensor networks. Proceedings of the 18th IEEE International Conference on Network Protocols.

[10] Vieira L.F.M., Kong J., Lee U., Gerla M. Analysis of Aloha Protocols for Underwater Acoustic Sensor Networks. https://www.semanticscholar.org/paper/Analysis-of-Aloha-Protocols-for-Underwater-Acoustic-Vieira-Kong/095862fcbbbde81ba0cbb51831276e8c0e70accb.

[11] Yackoski J., Shen C.C. UW-FLASHR: Achieving High Channel Utilization in a Time-Based Acoustic Mac Protocol. Proceedings of the 3rd International Workshop on Underwater Networks.

[12] Hsu C.C., Lai K.F., Chou C.F., Lin K.C.J. ST-MAC: Spatial-Temporal MAC Scheduling for Underwater Sensor Networks. Proceedings of the IEEE INFOCOM 2009.

[13] Kredo II K., Djukic P., Mohapatra P. STUMP: Exploiting Position Diversity in the Staggered TDMA Underwater MAC Protocol. Proceedings of the IEEE INFOCOM 2009.

[14] Zhuo X., Qu F., Yang H., Wei Y., Wu Y., Li J. (2019). Delay and Queue Aware Adaptive Scheduling- Based MAC Protocol for Underwater Acoustic Sensor Networks. IEEE Access.

[15] Peleato B., Stojanovic M. (2007). Distance aware collision avoidance protocol for ad-hoc underwater acoustic sensor networks. IEEE Commun. Lett..

[16] Molins M., Stojanovic M. Slotted FAMA: A MAC protocol for underwater acoustic networks. Proceedings of the OCEANS 2006–Asia Pacific.

[17] Ng H.H., Soh W.S., Motani M. MACA-U: A Media Access Protocol for Underwater Acoustic Networks. Proceedings of the IEEE GLOBECOM 2008–2008 IEEE Global Telecommunications Conference.

[18] Guo X., Frater M.R., Ryan M.J. (2009). Design of a Propagation-Delay-Tolerant MAC Protocol for Underwater Acoustic Sensor Networks. IEEE J. Ocean. Eng..

[19] Abramson N. The ALOHA SYSTEM: Another Alternative for Computer Communications. Proceedings of the Fall Joint Computer Conference.

[20] Chirdchoo N., Soh W.S., Chua K.C. Aloha-Based MAC Protocols with Collision Avoidance for Underwater Acoustic Networks. Proceedings of the IEEE INFOCOM 2007–26th IEEE International Conference on Computer Communications.

[21] Syed A.A., Ye W., Heidemann J., Krishnamachari B. Understanding Spatio-Temporal Uncertainty in Medium Access with ALOHA Protocols. Proceedings of the 2nd Workshop on Underwater Networks.

[22] Zhou Y., Chen K., He J., Guan H. Enhanced Slotted Aloha Protocols for Underwater Sensor Networks with Large Propagation Delay. Proceedings of the 2011 IEEE 73rd Vehicular Technology Conference (VTC Spring).

[23] Syed A.A., Ye W., Heidemann J. T-Lohi: A New Class of MAC Protocols for Underwater Acoustic Sensor Networks. Proceedings of the IEEE INFOCOM 2008–The 27th Conference on Computer Communications.

[24] Jin L., Huang D.D. (2013). A slotted CSMA based reinforcement learning approach for extending the lifetime of underwater acoustic wireless sensor networks. Comput. Commun..

[25] Ahmed F., Cho H.S. (2021). A Time-Slotted Data Gathering Medium Access Control Protocol Using Q-Learning for Underwater Acoustic Sensor Networks. IEEE Access.

[26] Cho J., Ahmed F., Shitiri E., Cho H.S. Power Control for MACA-based Underwater MAC Protocol: A Q-Learning Approach. Proceedings of the 2021 IEEE Region 10 Symposium (TENSYMP).

[27] Gazi F., Ahmed N., Misra S., Wei W. (2022). Reinforcement Learning-Based MAC Protocol for Underwater Multimedia Sensor Networks. ACM Trans. Sen. Netw..

[28] Alhassan I.B., Mitchell P.D. (2021). Packet Flow Based Reinforcement Learning MAC Protocol for Underwater Acoustic Sensor Networks. Sensors.

[29] Park S.H., Mitchell P.D., Grace D. Performance of the ALOHA-Q MAC Protocol for Underwater Acoustic Networks. Proceedings of the 2018 International Conference on Computing, Electronics and Communications Engineering (iCCECE).

[30] Ye X., Yu Y., Fu L. (2022). Deep Reinforcement Learning Based MAC Protocol for Underwater Acoustic Networks. IEEE Trans. Mob. Comput..

[31] Liu E., He R., Chen X., Yu C. (2022). Deep Reinforcement Learning Based Optical and Acoustic Dual Channel Multiple Access in Heterogeneous Underwater Sensor Networks. Sensors.

[32] Roberts L.G. (1975). ALOHA Packet System with and without Slots and Capture. ACM SIGCOMM Comput. Commun. Rev..

[33] (2022). EvoLogics. https://evologics.de/acoustic-modem/18-34.

[34] Caruso A., Paparella F., Vieira L.F.M., Erol M., Gerla M. The Meandering Current Mobility Model and its Impact on Underwater Mobile Sensor Networks. Proceedings of the IEEE INFOCOM 2008–The 27th Conference on Computer Communications.

